# The 3-Second Rule in Hereditary Pure Cerebellar Ataxia: A Synchronized Tapping Study

**DOI:** 10.1371/journal.pone.0118592

**Published:** 2015-02-23

**Authors:** Shunichi Matsuda, Hideyuki Matsumoto, Toshiaki Furubayashi, Ritsuko Hanajima, Shoji Tsuji, Yoshikazu Ugawa, Yasuo Terao

**Affiliations:** 1 Department of Neurology, The University of Tokyo, Tokyo, Japan; 2 Department of Neurology, School of Medicine, Fukushima Medical University, Fukushima, Japan; Centre de Neuroscience Cognitive, FRANCE

## Abstract

The ‘3-second rule’ has been proposed based on miscellaneous observations that a time period of around 3 seconds constitutes the fundamental unit of time related to the neuro-cognitive machinery in normal humans. The aim of paper was to investigate the temporal processing in patients with spinocerebellar ataxia type 6 (SCA6) and SCA31, pure cerebellar types of spinocerebellar degeneration, using a synchronized tapping task. Seventeen SCA patients (11 SCA6, 6 SCA31) and 17 normal age-matched volunteers participated. The task required subjects to tap a keyboard in synchrony with sequences of auditory stimuli presented at fixed interstimulus intervals (ISIs) between 200 and 4800 ms. In this task, the subjects required non-motor components to estimate the time of forthcoming tone in addition to motor components to tap. Normal subjects synchronized their taps to the presented tones at shorter ISIs, whereas as the ISI became longer, the normal subjects displayed greater latency between the tone and the tapping (transition zone). After the transition zone, normal subjects pressed the button delayed relative to the tone. On the other hand, SCA patients could not synchronize their tapping with the tone even at shorter ISIs, although they pressed the button delayed relative to the tone earlier than normal subjects did. The earliest time of delayed tapping appearance after the transition zone was 4800 ms in normal subjects but 1800 ms in SCA patients. The span of temporal integration in SCA patients is shortened compared to that in normal subjects. This could represent non-motor cerebellar dysfunction in SCA patients.

## Introduction

Estimating brief time periods is considered essential to a variety of cognitive and mental processes [1, 2]. It has been suggested that there is a limit to the span of time that one can integrate and perceive as a ‘perceptual unit’, which has been supported by a number of studies on the temporal reproduction of stimuli [3]. For example, whereas stimuli are reproduced relatively accurately up to 3 seconds in temporal reproduction tasks, longer stimuli are incorrectly reproduced [4]. These studies led to the idea that there is a limit to a time window within which the central nervous system can integrate into a perceptual unit, which would be considered to represent a ‘subjective present’. The limit of temporal processing is considered to lie somewhere around 3 seconds [5]. The ‘3-second rule’ has been proposed based on miscellaneous observations that a time period of around 3 seconds constitutes the fundamental unit of time related to the neuro-cognitive machinery in higher mammals including normal humans [3].

Although at present no consensus exists as to the precise role of the cerebellum in timing function, various behavioral studies have suggested that cerebellar input and output flow is important for processing time information, and that the cerebellum is important for the neural representation of time [6–9]. A wide variety of imaging studies have also shown activation of the cerebellum during the temporal discrimination of short intervals [10–14]. However, the role of the cerebellum in temporal processing remains to be clarified. Ivry proposed that the role of the cerebellum in temporal processing is specialized for the sub-second range, whereas the basal ganglia process timing in the seconds to minutes range [15]. Recently it has been shown that the neural mechanisms in the temporal processing differ between the sub-second range and the supra-second range [16].

If this hypothesis is correct, we would predict that the span of temporal integration in the range of 2–3 seconds is not affected in cerebellar patients. Therefore, we investigated the 3-second rule in patients with spinocerebellar ataxia (SCA) presenting with pure cerebellar symptoms using a synchronized tapping task which required non-motor components to estimate the time of forthcoming tone in addition to motor components to tap [17, 18]. However, it turned out that the span of temporal integration was shortened in cerebellar disorders.

## Materials and Methods

### Patients and control subjects

Seventeen non-demented spinocerebellar degeneration (SCD) patients and 17 age-matched normal subjects participated in the study. Eleven SCA patients were genetically confirmed as spinocerebellar ataxia type 6 (SCA6) and six as spinocerebellar ataxia type 31 (SCA31). The patients with SCA6/31 were an autosomal dominant cerebellar atrophy caused by a CAG trinucleotide/TGGAA pentanucleotide repeated expansion on chromosome 19p13/16q22 [19–22]. The severity of ataxia in each subject was rated on ICARS [23]. The Mini–Mental Status Examination (MMSE) [24] was used to exclude SCA patients and normal subjects with severe dementia from this study. The age of SCA patients was 65.0 ± 11.0 (mean ± standard deviation) years (range: 43–81 years) and that of normal subjects was 63.0 ± 11.0 years (range: 42–79 years). There were no statistically significant difference in age between the two subject groups (Mann–Whitney U test, P = 0.890). The median MMSE score of SCA patients was 29 (range 26–30), and that of normal subjects was 30 (range: 27–30). There was no significant difference between the two groups (Mann–Whitney U test, P = 0.068). The median duration of illness in SCA patients was 7 years (range: 3–27 years). The median ICARS score of SCA patients was 54 (range: 5–73). We excluded subjects with cognitive or sensory impairment, or other neurological disorders.

Written informed consent to participate in this study was obtained from all subjects. The procedure was approved by the Ethics Committee of The University of Tokyo, and the study was conducted in accordance with the ethical standards of the Declaration of Helsinki.

### Synchronized tapping task

**Fig 1:**
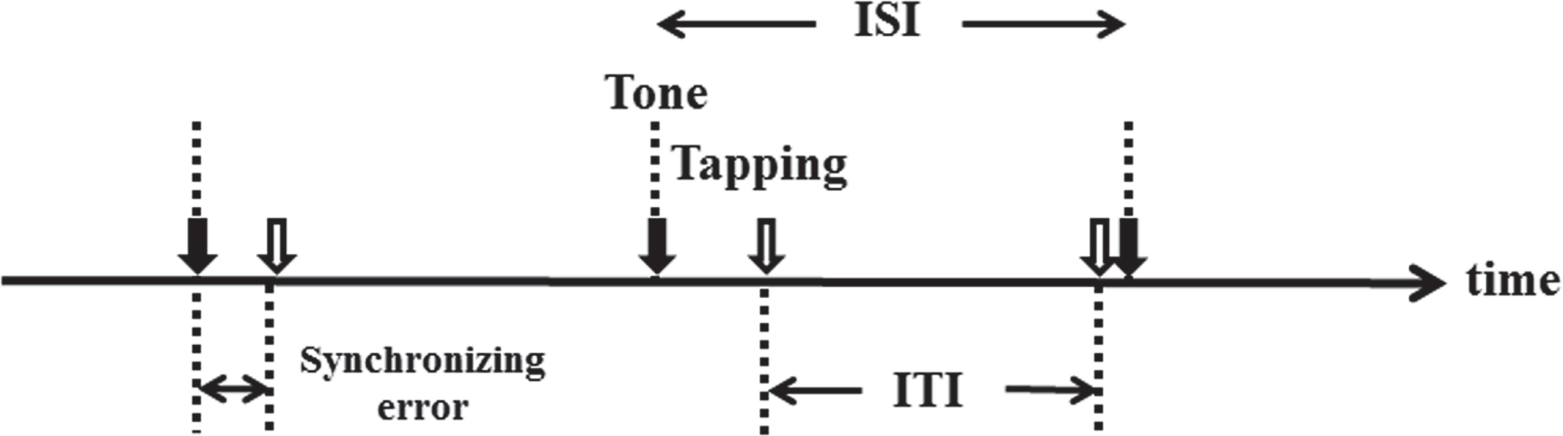
Synchronized tapping task. The subjects tapped the keyboard in synchrony with the sequences of auditory tones presented at fixed interstimulus intervals (ISIs) between 200 and 4800 ms. We measured the time difference (synchronizing error) between the times of tap (white arrows) and those of tones (black arrows) and the time interval between successive taps (ITI) in each trial.

The synchronized tapping task used in the present study is depicted in Fig. 1. The program for the experiment was implemented using commercial software (Experiment Builder, SR Research Ltd., Ontario, Canada), which allows both presentation of stimuli (in this case, a tone sequence) and online collection of the performance data (the time of button press made by the subjects) for further analysis. A space key on the computer keyboards was used as the response key. The key produced no additional sound when pressed.

The subject was instructed to tap the space bar of the keyboard with subject’s right index finger in synchrony with the tones presented at a fixed interval through a speaker as precisely as possible. In each session, a sequence consisting of 110 tones was presented. Although no training or feedback was provided, all the subjects understood the task correctly within the first 10 taps. ISI in each sequence was kept at a constant value (isochronous sequence), which was selected from among eight different ISIs according to the methods of Mates et al. [17]: 200, 250, 333, 500, 600, 900, 1200, 1800, 2400, 3600, and 4800 ms. The order of presentation of the sequences with different ISIs was randomized across subjects, until each subject completed the sessions for all ISIs. Each tone had a frequency of 500 Hz and duration of 50 ms. Since the tones were presented at a regular pace, subjects were required to predict the time of the forthcoming tone and to synchronize the tap with the onset of each tone. Rhythmic stimulus templates presented were quickly acquired by the subjects, and synchronization with repetitive rhythmic patterns was usually established within several taps.

### Simple reaction task

In a separate session, we assessed the simple reaction time (SRT) to a single tone; the subject was instructed to press the space bar as quickly as possible after listening to the tone. Total 110 tones were presented at the interval of 4800 ms. This measure allowed us to study how much time it took for the subjects to press the space bar just after the presentation of auditory stimuli, reflecting simple somatomotor functions in SCA patients and normal subjects.

### Data collection

The data collected were the time of keyboard press and the time of tone presentation. The first 10 taps in a sequence were discarded from the analysis until the subjects usually adapted to the sequence of regular tones within several tones (see above), and the remaining 100 responses were recorded from each session. From these data, we derived the time difference between each tap onset and stimulus onset (synchronizing error), and the time interval between successive taps (intertap interval, ITI) according to the previous paper [17]. Synchronizing error was calculated as follows: synchronizing error = tap onset – stimulus onset, where positive values of synchronizing error indicated the taps delayed relative to the tone, while negative values implied the taps preceding the tone. In the simple reaction task, the SRT was measured.

We constructed a histogram depicting the distribution of synchronizing error for 100 trials in a session for a subject at each ISI. Typically in normal subjects, at shorter ISIs, the histogram of synchronizing error showed a unimodal distribution, whereas when ISI became longer and exceeded a certain value, it showed a broader distribution (transition zone). The authors in the previous study recognized a bimodal distribution rather than this kind of broad distribution [17]. Here, we used the term “broader” distribution instead of “bimodal” distribution, because during the transition, the histogram frequently exhibited a broader distribution without necessarily forming distinctly bimodal distribution peaks. This was true both for the histograms of the Mates’ and our studies. After the time of broad distribution, the histogram again returned to a unimodal distribution [17]. Therefore, we termed the time showing a unimodal distribution after the transition zone the time at which delayed tapping appeared (delayed tapping time) in the distribution of synchronizing error.

Although we observed a transition zone in all normal subjects (see Results), it was often the case that the transition zone from unimodal to broader distribution was not as clear-cut as reported previously [17], especially in SCA patients (see Results). Therefore, to compare the distributions of synchronizing error in SCA patients with those in normal subjects, we compared the SD of synchronizing error between SCA patients and normal subjects. We also measured the time at which delayed tapping appeared instead of the time of transition zone in both SCA patients and normal subjects. To measure the delayed tapping time, we calculated the synchronizing error at each ISI and determined which ISI became significantly different from 0 ms.

### Statistical assessment

The following statistical analyses were performed using the SPSS software package (version 19.0; SPSS Inc., Chicago, IL). (i) To compare SRT between SCA patients and normal subjects, the unpaired-*t* test was used. (ii) To assess how accurately the subjects reproduced ISIs of the presented tone sequence by tapping, the ratio of average ITI to ISI at each ISI was calculated for both two groups. This parameter was analyzed using a two-way analysis of variance (ANOVA) with repeated measures, with a within-subject factor: ISI and a between-subject factor: subject group. Where necessary, the Greenhouse-Geisser correction was used to evaluate non-sphericity. Post-hoc analyses were also conducted, if necessary, using the unpaired-t test (between-subject factor). (iii) To evaluate the variation of synchronizing tapping, the SD of synchronizing error at each ISI was calculated for both groups. This parameter was also analyzed using a two-way analysis of variance (ANOVA) with repeated measures. (iv) To measure the time at which delayed tapping appeared, the Mann-Whitney's U test was used to study whether the median of the individual synchronizing error deviated significantly from 0 ms or not at each ISI in both groups. (v) To investigate influence of the cerebellar motor dysfunction (ataxia) on the tapping in SCA patients, Spearman's rank correlations between tapping parameters and ICARS were done in SCA patients.

## Results

### Simple reaction task

SRT in SCA patients was significantly longer than that in normal subjects (SCA patients: 379.3 ± 114.0 ms; normal subjects: 266.7 ± 66.3 ms, *P* = 0.002). This suggested that SCA patients were unable to tap the button as quickly as normal subjects.

### Accuracy of tapping

**Fig 2:**
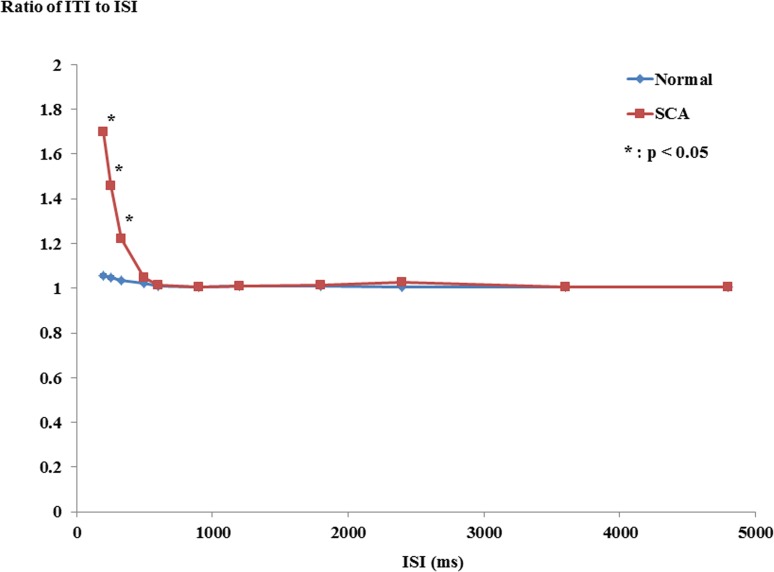
The ratio of ITI to ISI. The ratio of average ITI to ISI is plotted in each ISI. The red curve is for SCA patients and the blue curve for normal subjects. Asterisks indicate significant difference between the two groups.

Fig. 2 plots the ratio of average ITI to ISI at each ISI for SCA patients and normal subjects. ANOVA revealed that this ratio decreased with increasing ISIs for both groups, especially at shorter ISIs (test of within-subject effect: ISI, *F*_1.065_ = 18.392, *P* < 0.001), whereas after around ISIs of 1 second it leveled off. A significant interaction was noted between ISI and subject group (test of within-subject effect: ISI × subject group interaction, *F*_1.065_ = 13.529, *P* = 0.001), which was because the ratio increased only in SCA patients, especially at shorter ISIs from 200 to 500 ms. Post-hoc analysis revealed that the ratio in SCA patients was significantly larger than that in normal subjects at shorter ISIs: 200 ms (*P* = 0.001), 250 ms (*P* = 0.005), 333 ms (*P* = 0.011). The results suggested that SCA patients could not reliably follow these fast paces of repetitive tones (ISIs 200, 250, and 333 ms).

### Inaccurate tapping in SCA patients

The ratios of average ITI to ISI at 200, 250, and 333 ms in SCA patients was higher than those in normal subjects, suggesting inaccurate tapping at shorter ISIs in SCA patients. Spearman's rank correlation showed a significant positive correlation between the sum of ratios of average ITI to ISI at ISI of 200, 250, and 333 ms and ICARS (*P* = 0.036, r = 0.512), suggesting the contribution of cerebellar motor dysfunction (ataxia) to inaccurate tapping at ISI of 200–333 ms in SCA patients. Thus, the data for these ISIs were excluded from the analysis of the time at which delayed tapping appeared.

### Synchronizing error

**Fig 3:**
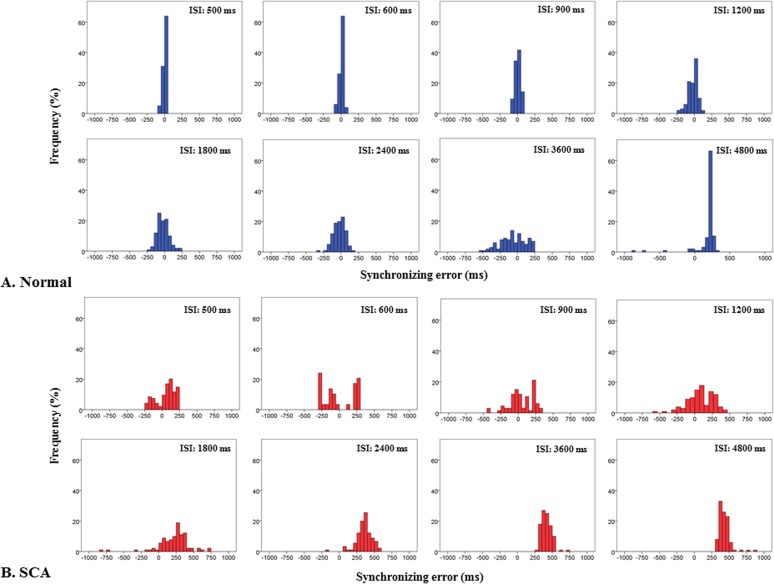
Representative distributions of synchronizing error. Distributions of synchronizing error (the time of button responses relative to the tone) are depicted as histograms in a normal subject (A) and a SCA patient (B). In a normal subject, at shorter ISIs, the histogram for synchronizing error shows a unimodal distribution which was centered around or a slightly negative value, whereas when ISI becomes more variable at intermediate ISIs (transition zone), it finally converges into a unimodal distribution delayed relative to the tone. In SCA patients, however, the unimodal distributions at shorter ISIs are not observed. Additionally, the earliest time of delayed tapping appears earlier compared to normal subjects. Consequently, the temporal processing is disrupted and the ‘3 second rule’ is shortened in SCA patients.

The time of button press relative to the time of tone presentation showed a relatively narrow unimodal distribution up to a certain ISI in normal subjects (Fig. 3A), which was largely clustered around a value around 0 ms or slightly negative. As the ISI increased, the distributions of the subjects’ button press relative to the tone gradually came to show a larger variability with both delayed and advanced responses relative to the tones. Finally, at longer ISIs, the distribution again came to take a unimodal distribution, but this time consisting mainly of the delayed responses, distinct from the early distribution clustered around 0 ms. The delayed unimodal distribution at 4800 ms took a clearly distinct distribution from that clustered around 0 ms. For the SCA patients (Fig. 3B), the early unimodal distribution was unclear and the delayed tapping time appeared to occur earlier (from 1800 to 4800 ms) than in normal subjects (4800 ms). Consequently, for these SCA patients, tapping failed to be in synchrony with tones from the beginning; the temporal processing came to be disrupted from shorter ISIs. Otherwise, the final transition to unimodal distribution of delayed responses was preserved and this delayed tapping time occurred earlier for these SCA patients than in normal subjects.

### Variability of synchronizing error

**Fig 4:**
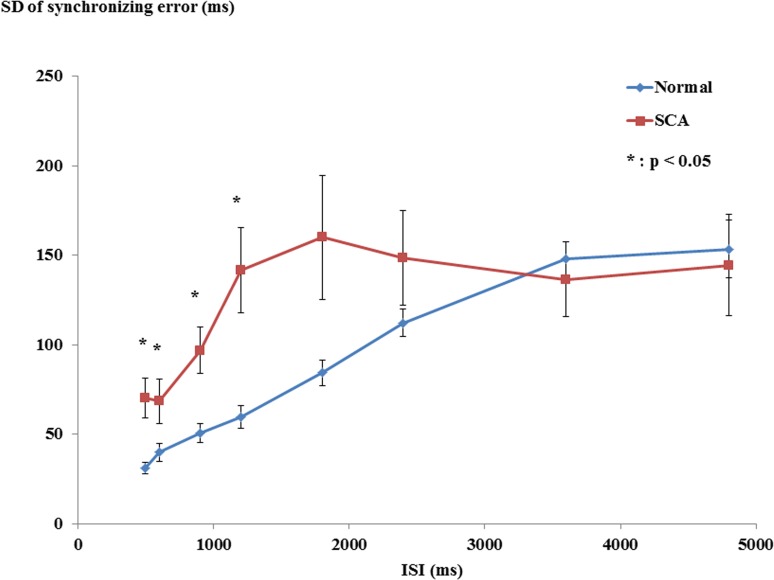
SD of synchronizing error. The SD of synchronizing error at each ISI are shown. The SD of synchronization error in SCA patients was significantly larger than that of normal subjects at ISIs 500–1200 ms, but was comparable at ISIs 1800–4800 ms. This suggests that the synchronizing tapping in SCA patients was disrupted at shorter ISIs (500–1200 ms).

To investigate the variability of synchronizing error, the SD of synchronizing error at each ISI was compared between SCA patients and normal subjects (Fig. 4). ANOVA revealed a significant interaction between ISI and subject group (ISI × subject–group interaction, F_3.513_ = 2.72, *P* = 0.040). Post-hoc analysis showed that the SD of synchronization error in SCA patients was significantly larger than that of normal subjects at ISIs 500–1200 ms (ISI 500 ms, *P* = 0.004; ISI 600 ms, *P* = 0.047; ISI 900 ms, *P* = 0.004; ISI 1200 ms, *P* = 0.004), but was comparable at ISIs 1800–4800 ms (ISI 1800 ms, *P* = 0.052; ISI 2400 ms, *P* = 0.214; ISI 3600 ms, *P* = 0.641; ISI 4800 ms, *P* = 0.794). The SD of synchronizing error increased for both groups with increasing ISI, during which it was greater in SCA patients than in normal subjects. This suggests that the accuracy of synchronized tapping in SCA patients was disrupted at shorter ISIs (500, 600, 900 and 1200 ms).

### Disrupted synchronizing tapping in SCA patients

To investigate why the synchronizing tapping was disrupted at the shorter ISIs (500, 600, 900 and 1200 ms) in SCA patients, we classified all ISIs into two ISI groups, the ISIs with and without disrupted synchronizing tapping (the former ISIs are 500, 600, 900 and 1200 ms and the latter ISIs 1800, 2400, 3600 and 4800 ms). We calculated the sum of SD of synchronizing error at the shorter ISIs (500, 600, 900 and 1200 ms) and those at the longer ISIs (1800, 2400, 3600 and 4800 ms) as global parameters of tapping variability at shorter and longer ISIs with and without disrupted synchronizing tapping, respectively. Spearman's rank correlation showed a significant positive correlation between the sum of SD of synchronizing error at ISI of 500, 600, 900 and 1200 ms and ICARS (*P* < 0.001, r = 0.789), suggesting the contribution of cerebellar motor dysfunction (ataxia) to disrupt synchronized tapping at these ISIs in SCA patients. On the other hand, Spearman's rank correlation showed no significant correlation between the sum of SD of synchronizing error at ISIs of 1800, 2400, 3600 and 4800 ms and ICARS (*P* = 0.406, r = 0.216), suggesting that cerebellar motor dysfunction may not contribute to the tapping around these ISIs in SCA patients.

### Delayed tapping time after transition zone

At a certain ISI, both normal subjects and SCA patients came to press the button after the tone presentation instead of synchronizing with the tone (Fig. 3). Table 1 shows the values of synchronizing error. We measured the earliest time of delayed tapping. Mann-Whitney's U test revealed that delayed tapping appeared at 1800 ms in SCA patients and 4800 ms in normal subjects (SCA patients: 500 ms *P* = 0.001, 600 ms *P* = 0.029, 900 ms *P* = 0.754, 1200 ms *P* = 0.348,1800 ms *P* = 0.029, 2400 ms *P* = 0.029, 3600 ms *P* < 0.001, 4800 ms *P* < 0.001; normal subjects: 500 ms *P* = 0.754, 600 ms *P* = 0.754, 900 ms *P* = 0.118, 1200 ms *P* = 0.118, 1800 ms *P* = 0. 348, 2400 ms, *P* = 0.348, 3600 ms *P* = 0.348, 4800 ms *P* = 0.029). Again, as already described, Spearman's rank correlation showed no significant correlation between the sum of SD of synchronizing error at ISIs from 1800 to 4800 ms and ICARS. These results suggested that the span of the temporal integration was shorter in SCA patients than in normal subjects, which should not be related to the cerebellar motor dysfunction (ataxia).

**Table 1:** The synchronizing error.

ISI	500 ms	600 ms	900 ms	1200 ms	1800 ms	2400 ms	3600 ms	4800 ms
SCA patients	18.8 ± 58.5	2.7 ± 68.2	-28.9 ± 100.5	1.0 ± 142.4	79.3 ± 214.1*	146.9 ± 214.3*	263.0 ± 192.6*	283.1 ± 193.5*
Normal subjects	3.4 ± 34.1	-10.2 ± 45.2	-29.8 ± 52.5	12.5 ± 30.9	-7.6 ± 83.0	-17.4 ± 94.9	9.8 ± 111.0	63.5 ± 132.1*

Data are shown in mean ± standard deviation

*significant deviation from 0 ms after transition zone (delayed tapping time)

## Discussion

In this study, we showed that the span of temporal integration which is considered to lie normally around 3s (the ‘3 second rule’) is shortened in pure cerebellar SCA patients; the earliest ISI where delayed tapping appeared in the distribution of response time relative to the tones was 1800 ms in SCA patients, significantly shorter ISIs than in normal subjects at 4800 ms. In the following, we discuss how temporal integration is affected in SCA patients, which may represent one of the “non-motor” functions subserved by the cerebellum.

### Impaired synchronized tapping in SCA patients at the shorter ISIs (200–333ms)

Impaired synchronized tapping in SCA patients was noted at shorter ISIs (200–333ms). As shown in Fig. 3, SCA patients were not able to attain rapid rates of sensorimotor synchronization (ISIs 200–333 ms). For these fast paces of tapping, sensory and/or motor limits due to cerebellar ataxia may play a primary role in the increased variability of tapping [18]. SRT in SCA patients was around 380 ms and that in normal subjects was around 270 ms (the difference is more than 100 ms), indicating the difficulty of fast response even to a single auditory stimuli. The variability of ITI at ISIs between 200–333 ms showed positive correlations with ICARS. This correlation suggested that the impaired tapping performance at shorter ISIs (200–333 ms) were at least partially due to cerebellar motor dysfunction, i.e. cerebellar ataxia.

### How does cerebellar dysfunction disrupt temporal integration in the sub- and supra-second range?

Before conducting this study, we predicted that the span of temporal integration in the range of 2–3 seconds would not be affected in cerebellar patients. As shown in Fig. 3 and Table 1, however, the tapping times relative to the presented tones showed distinct distributions at ISIs shorter and longer than the transition in both subject groups. In normal subjects, the distribution for shorter ISIs (500–1200 ms) clustered around 0 ms or slightly negative and that for longer ISIs (4800 ms) clustered apart from 0 ms, whereas in SCA patients, the unimodal distribution for shorter ISIs (500–1200 ms) were disrupted and that for longer ISI (1800–4800 ms) clustered apart from 0 ms. Thus, although SCA patients were more variable in their time of button press, presumably reflecting cerebellar ataxia, they were able to predict the times of forthcoming tones at shorter ISIs to some extent and to press the button roughly in synchrony with them. In contrast, as the ISI became longer, it was no longer possible for both subject groups to predict the time of forthcoming tone and the button presses became delayed with respect to the tones. In particular, the synchronized tapping at ISIs between 1800–4800 ms cannot be explained solely on the basis of cerebellar ataxia, which showed no correlation with ICARS. Thus, in contrast to our prediction, we have to consider temporal processing in the sub-second range at shorter ISIs and supra-second range at longer ISIs separately. Our findings are compatible with the consideration of temporal integration proposed by the previous paper of Mates et al. [17]: when the temporal integration capacity is overrun, SCA patients are in a situation of reaction to the stimuli as well as normal subjects.

### Neural structures responsible for the shortened temporal integration in SCA patients

Distinct neural systems have been postulated to subserve time estimation of long and short durations; the ‘automatic’ or ‘bottom up’ system, processing time mainly in the sub-second (millisecond) range, draws on motor circuits, including the cerebellum, whereas the ‘cognitively controlled’ or ‘top down’ system processing time in the supra-second range depends on prefrontal and parietal cortical regions as well as the basal ganglia [12, 15, 25]. Based on this notion, we expected that cerebellar dysfunction would be associated with the impaired temporal processing in the sub- but not in the supra-second range. However, our results showed that the span of temporal processing in the second range was shortened, which would correspond to the impairment of ‘cognitively controlled’ timing system. Indeed, recent studies suggest that the cerebellum may be involved also in temporal processing in the range of 2–3 seconds, which should involve attention in addition to motor control itself [7, 12].

What could be the consequences of the cerebellar lesions we studied here and how do they lead to the disruption of temporal integration? Several neural structures have been implicated in the shortening of temporal integration. The first possibility that the dysfunction of frontal midline cortical regions, which are considered to serve perceptual coding of interval time in the seconds range both in primates [26] and in humans [27, 28], may mainly contribute to the shortened temporal integration in SCA patients. Coding of interval time has been associated with contingent negative variation (CNV), whose main generator is located over midline frontal areas including the anterior and posterior supplementary motor areas [28–31]. Compared with normal subjects, the late CNV is significantly smaller or absent in patients with Parkinson’s disease [32], in whom various temporal judgment deficits have been reported. On the other hand, a case report showed that CNV is normal in a cerebellar patient [33]. While this mechanism is plausible, we consider it unlikely that the shortening of temporal integration in SCA patients can be solely ascribed to dysfunction of midline cortical structures.

Another possible explanation may be that the synchronized tapping may be implemented in a network-like manner through the cerebello-diencephalic-parietal loop. As noted above, in order to perform the present synchronized tapping task, the cerebellum may predict in a feedforward manner the time points of button press and at which the sensory outcome of movement would occur. Cerebro-cerebellar interactions are shown to underlie such feedforward computation [34, 35]. Indeed, a magnetoencephalographic recording during the performance of a synchronized tapping task at an ISI of 800 ms demonstrated two sources localized in the contralateral primary motor cortex approximately 77 ms before tap onset, and over the contralateral sensory cortex 75 ms after tap onset [36]. Subjects may also use sensory feedback through the cerebello-diencephalic-parietal loop, including the inferior parietal lobule (IPL), in judging and evaluating whether they are ‘keeping track of time’, while parietal-cerebellar interaction may be also critical for feedback processing [37]. Functional disconnections between the parietal cortex and the cerebellum would thus compromise sensorimotor synchronization at longer ISIs by disruption of this parietal-cerebellar interaction involved in attention and working memory [25].

As a third explanation, Morillon et al. (2009) suggested that whereas the motor system including the cerebellum automatically tracks durations below 2 s, mesial brain regions of the so-called default mode network subserve processing of durations above 2 s as studied in the present research [16]. In this case, disruption of temporal processing may be mediated by the dysfunction of the default mode network, including IPL, which has a functional connection with cerebellar lobule IX and vermal VIIIb [38]. In this context, Hayashi et al. (2014) suggested that supra-second durations are processed in the parietal cortex by utilizing the capacity of attention and working memory to keep track of time [25].

Note that the three explanations above are not mutually exclusive, in that all implicate both the cerebellum and the midline frontal regions. The first explanation is plausible in view of the functional connections between the medial frontal regions and the cerebellum. Cerebello-diencephalic-parietal loop referred to in the second possible mechanism would include projections possibly from midline frontal regions such as the supplementary motor area to the cerebellum through the cortico-ponto-cerebellar pathway. Finally, the default mode network raised as the third possibility includes medial frontal regions with connections with the cerebellum. Although we can assume other possible explanations, further investigations in the other disorders may be required to elucidate the neural mechanisms responsible for the shortened temporal integration in SCA patients.

Enlarged window of temporal processing (longer ‘now’) has been reported in patients with schizophrenia. Subjectively, this can lead to impaired duration perception, and general inability to organize time, including loss of subjective continuity and subjective fragmentation of the world such as the temporal fragmentation, perturbed discrimination of simultaneous and synchronous events as well as abnormal perceived simultaneity of desynchronized audiovisual speech in these patients [39]. In contrast, what kind of subjective perception the shortened window of temporal integration in cerebellar patients can lead to is entirely unknown and is a theme for our ongoing investigation [3, 40].

### Future studies of temporal integration based on the issues in this study

There are several limitations in this study, which would need to be addressed in future studies. First, the data obtained by tapping a keyboard may not be sensitive enough for evaluating the small timing differences because of the low temporal resolution. In future studies, therefore, a device with the higher temporal resolution should be used. Another issue is that, for evaluating SRT in the simple reaction task, we did not use randomized ISIs due to limitations in the computer programs, but instead the different instructions were given for the simple reaction task and synchronizing tapping task. In future studies, however, SRT should be measured using randomized ISIs. Finally, it is well known that both age and neurological disorder influence SRT and the variance and the effects of these factors should also be taken into account [41]. To investigate whether age and types of SCA influence the parameters related to temporal integration, i.e. SRT, synchronizing error, and SD of synchronizing error, we performed the following regression analyses. However, the analysis for normal subjects showed no correlation of age with the parameters. Similarly, the analysis for SCA patients showed no correlation of age or type of SCA with the parameters (unpublished data). However, the negative data may derive from the small number of the participants in this study. In future studies, we expect to reveal relationships between age and temporal integration in a larger scale research.

## Conclusions

Using the synchronized tapping task, we showed that the span of temporal integration is shortened in SCA patients. The shortening of the span of temporal integration in SCA patients may be one of the manifestations of non-motor cerebellar dysfunction.
